# An All Fiber Intrinsic Fabry-Perot Interferometer Based on an Air-Microcavity

**DOI:** 10.3390/s130506355

**Published:** 2013-05-14

**Authors:** Daniel Jáuregui-Vázquez, Julián M. Estudillo-Ayala, Roberto Rojas-Laguna, Everardo Vargas-Rodríguez, Juan M. Sierra-Hernández, Juan C. Hernández-García, Ruth I. Mata-Chávez

**Affiliations:** 1 Optoelectrónica-CA, Departamento de Electrónica, DICIS, Universidad de Guanajuato, Carr. Salamanca-Valle km 3.5+1.8, Palo Blanco, Salamanca, Gto 36855, Mexico; E-Mails: julian@ugto.mx (J.M.E.-A.); rlaguna@ugto.mx (R.R.-L.); jm.sierrahernandez@ugto.mx (J.M.S.-H.); jucahdzga@hotmail.com (J.C.H.-G.); 2 Espintrónica y Óptica Aplicada-CA, Departamento de Estudios Multidiciplinarios, DICIS, Universidad de Guanajuato, Avenida Universidad s/n, Col. Yacatitas, Yuriria, Gto 38940, Mexico; E-Mails: evr@ugto.mx (E.V.-R.); ruth@ugto.mx (R.I.M.-C.)

**Keywords:** Fabry-Perot interferometer, microcavity, hollow core photonic crystal fiber, refractive index sensor, 07.60.Ly, 78.20.Ci

## Abstract

In this work an Intrinsic Fabry-Perot Interferometer (IFPI) based on an air-microcavity is presented. Here the air microcavity, with silica walls, is formed at a segment of a hollow core photonic crystal fiber (HCPCF), which is fusion spliced with a single mode fiber (SMF). Moreover, the spectral response of the IFPI is experimentally characterized and some results are provided. Finally, the viability to use the IFPI to implement a simple, compact size, and low cost refractive index sensor is briefly analyzed.

## 1. Introduction

Fiber optic interferometers have been investigated for a long period of time because of their potential application in several fields of science and technology [1–3], hence different interferometer fabrication techniques can be found in literature [4–7]. Among these of the most popular is the Fabry-Perot Interferometer (FPI) since it presents useful optical characteristics which allow us to measure different physical magnitudes such as pressure [5,8], strain [6], vibrations [6], temperature [9], refractive index (RI) [10,11] and magnetic field [12]. Recently, researchers have been developing different methods to fabricate intrinsic Fabry-Perot interferometers (IFPI) based on different techniques, for instance chemical etching [13], femtosecond laser micromachining [1] and by splicing different types of optical fiber using cleaving [14]. Most of these methods are shown to have high repeatability, which is quite important for sensing applications. However some of these fabrication methods can be difficult to implement since they can require a complex manufacturing process or even the use of noncommercial photonic crystal fibers. Hence, for its simplicity the fabrication method based on splicing two different optical fibers is an attractive option [15–19]. However, this method has some inherent challenges, for example it is necessary to create a fusion arc splicer program to correctly splice the optical fibers and to design an overall process to guarantee fabrication repetitiveness.

In this work an IFPI based on an air microcavity, which is fabricated within a segment of hollow core photonic crystal fiber (HCPCF), is presented. Here a standard arc fusion splicer was used to splice two different optical fibers (HCPCF and single mode fiber) and to form the air microcavity. Consequently, thermal effects can affect the properties of the physical joint [11] which can limit its performance. Therefore a fusion splicer program was created to control the arc discharges characteristics which allowed us to splice the fibers. Furthermore, to form the air microcavity another program for the fusion splicer was created. These programs and the overall IFPI fabrication method are described in this work. Moreover, the IFPI principle of operation is also discussed as well as some experimental results and its spectral response characterization are provided. Finally the viability to use the IFPI in the implementation of a simple and low cost refractive index sensor is briefly analysed.

## 2. IFPI Based on an Air Microcavity Fabrication Procedure

### 2.1. The Splice Joint SMF-HCPCF

**Figure 1.**
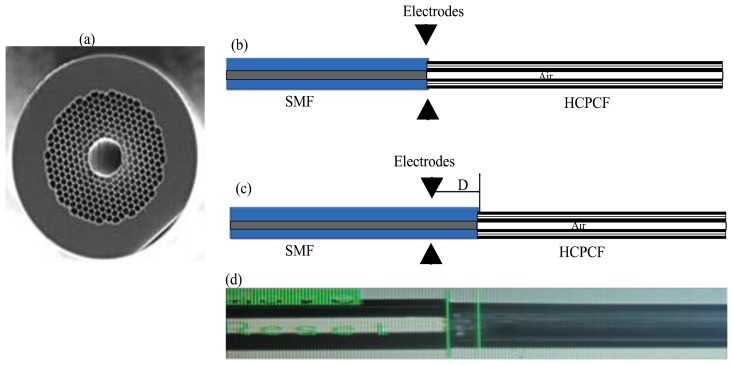
HCPCF-SMF splice joint; (**a**) HCPCF transversal view; (**b**) Initial position at the splicer; (**c**) Offset distance; and (**d**) Exampled of the splice joint obtained.

The fusion splicing of standard single mode fiber (SMF28) with a segment of HCPCF (Thorlabs HC-1060 19 Cells, Newton, NY, USA) is a compulsory step in the air microcavity fabrication, which is basically the main part of our IFPI. Here, it was necessary to form a splice joint with a non-collapsed region. In Figure 1(a) a transversal view photograph of the HCPCF used in our experiment is shown. The splice joint was achieved using a conventional splicer (Fitel-S175, Peachtree, GA, USA) and performing the following procedure: (a) Cut both optical fibers and set them at the initial position on the fusion splicer (see schematic in Figure 1(b)); (b) Horizontally displace both fibers up to a distance D = 80 μm from the electrodes, as shown in Figure 1(c); (c) Program the fusion splicer with the set of parameters listed in Table 1 (for clarity purposes let us to call this set of parameters Splicer Program 1); (d) Apply three arc discharges to form the splice joint (for a photograph see Figure 1(d)).

### 2.2. Air Microcavity Fabrication Procedure

**Figure 2.**
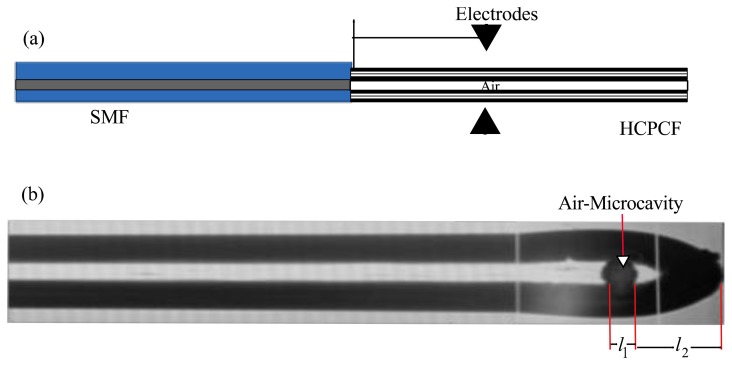
Air-Microcavity fabrication procedure: (**a**) Offset cut distance; (**b**) Example an IFPI fabricated using L = 200 μm and seven arc discharges.

After the splice joint with a non-collapsed region was formed, the next step consisted on the air microcavity fabrication. This was achieved by performing the following procedure: (a) Horizontally displace the splice joint a distance *L* from the fusion splicer electrodes (Figure 2(a)). Here it is important to point out that this displacement can be carried out by using the manual mode of the splicer without the need of removing the splice joint from the splicer; (b) Program the fusion splicer with the set of parameters listed in Table 1 (for clarity purpose let us to call this set of parameters Splicer Program 2); (c) Cut the HCPCF, at the offset distance *L* by applying seven arc discharges; (d) Apply the splicer clean process; (e) Apply the pre-fusion program of the fusion splicer. Here it is important to mention that one part of the HCPCF will collapse forming a silica wall and therefore a quantity of air will be confined between this wall and the SMF; (f) Apply another set of arc discharges to change the length of the air microcavity.

As can be appreciated in both procedures described above the arc fusion splicer is used only to form the splice joint and the air microcavity. However the fusion splicer alignment procedure was not used at all. Here the offset distance (*L*) and the number of arc discharges (*ND*) are some of the main parameters in the fabrication process and therefore it is important to characterize their effect over the final IFPI properties. In this way we fabricated some IFPI using different *L* distances to analyse its effects over the air microcavity fabrication process. From these experiments we found that for *L* < 100 μm the hollow core fiber completely collapsed, making impossible the air microcavity formation. Moreover, for *L* > 350 μm the HCPCF do not collapsed uniformly and therefore was not possible to form the air microcavity. For this reason and according to experimental results we considered for the IFPI fabrication an operating range of 100 < *L* < 350 μm.

## 3. Principle of Operation of the FPI Based on an Air Microcavity

**Figure 3.**
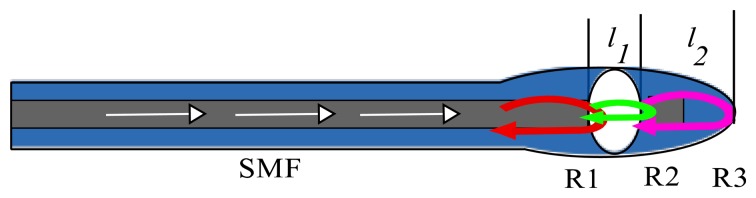
Schematic representation of the IFPI principle of operation.

The IFPI mirrors are formed by the boundaries between the air microcavity and the optical fibers (SMF and HCPCF). Since our IFPI will be physically formed at the tip of the SMF (see Figure 2(a)) therefore it will have silica mirrors. Consequently the IFPI spectrum will be mainly formed by the interference of three reflections (R1, R2 and R3) [1,17,18] because of the mirrors low reflectivity (see Figure 3). Here, the first reflection (R1) occurs when the light travelling inside the core of the SMF, which has a refractive index of *n*_1_ ≈ 1.4682, reaches the air microcavity. At this interface one part of the light is reflected (R1) and the rest is transmitted into the air microcavity. In this cavity the medium is air (*n*_2_ ≈ 1) and the light will travel a distance *l*_1_ until it reaches the silica wall, which was formed by the collapsed HCPCF cladding. At this interface a part of the light is reflected (R2) and the rest is transmitted into the silica wall with refractive index is *n*_1_ ≈ 1.4682 and thickness *l*_2_. Finally at the end of the silica wall another reflection (R3) will occur at the interface formed by the silica wall and the medium around the IFPI tip with refractive index *n*_3_.

At this point it is important to mention that at the splice joint some birefringence effects can be present since there are changes in the form of the propagation path and consequently some polarization changes can occur. However, this effect it is neither studied nor characterized in this work since we focused on the IFPI fabrication process and on the air microcavity characterization.

## 4. Characterization of the FPI with an Air Microcavity

### 4.1. Experimental Setup

**Figure 4.**
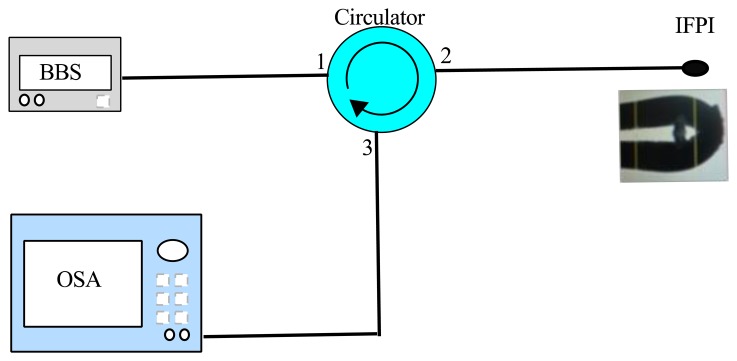
Setup for IFPI characterization.

To characterize the IFPI optical response the experimental setup shown in Figure 4 was used. Here the light from the partial-polarized broadband source (BBS) travels through an optical circulator (PICT-1550-S-*Z, Sumitomo Osaka Cement Co., LTD, Tokyo, Japan). Afterwards, the light reaches the IFPI and its reflection interference spectrum is monitored with an optical spectrum analyzer (Yokogawa, AQ6370B Co., LTD, Newnan, GA, USA).

### 4.2. Characterization of the IFPI Spectral Response Considering Different *L* Distances

**Figure 5.**
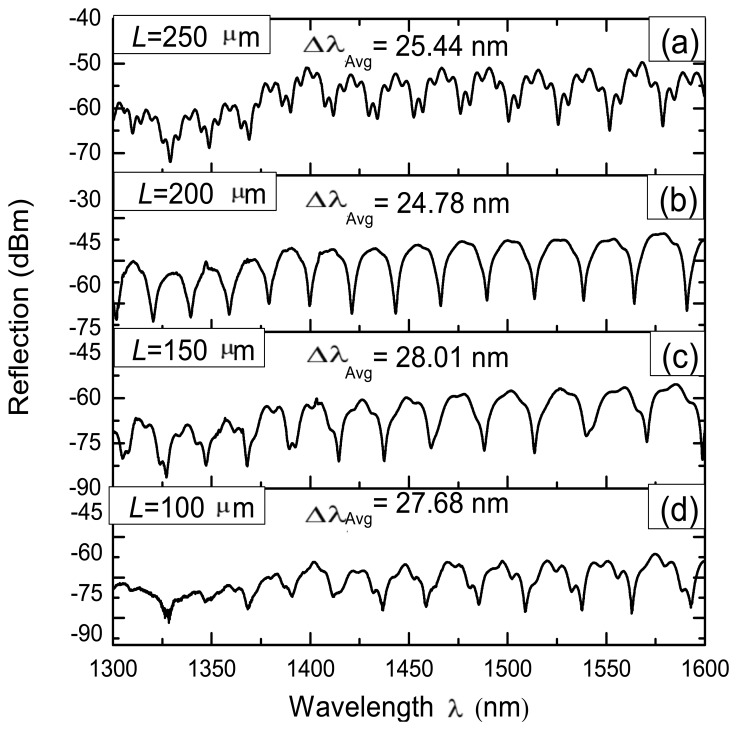
Measured IFPI reflection spectrum.

Some IFPIs considering different *L* distances were fabricated in order to characterize its effects over the spectral response. For practical reason we used *L* = 100, 150, 200 and 250 μm since these are within the operating range of 100 < *L* < 350 μm. Moreover, for all these IFPIs the same number of arc discharges (*ND* = 7) was used. Here during the fabrication of these IFPIs the length of the air microcavity was measured using the fusion splicer and in average obtained that this was approximately *l*_1_ = 48 μm. Moreover, also using the fusion splicer it was possible to measure the thickness of the silica wall and in average, for the four IFPIs, was approximately *l*_2_ = 70 μm. Using these values it is possible to determine the free spectral range of the IFPI fabricated by using the next formula [20]

(1) $$\Delta \lambda =\frac{{\lambda_{0}}^{2}}{2nl}$$

where *λ*_0_ is the reference wavelength, Δ*λ* is the spectral separation between two consecutive fringes, *n* is the refractive index of the FPI cavity (in our case is air with *n* = 1) and *l* is the air microcavity length (*l* = *l*_1_). Using these values we obtained that the free spectral range for our FPIs should be approximately Δ*λ* = 21.90 nm. In Figure 5 measured reflection spectra for these IFPIs are shown. In these graphs it is only presented the spectral range from 1,300 to 1,600 nm since it is the operating range of the optical circulator. From these spectra we determined in average Δ*λ* = 26.48 nm which is very close to the physically measured with the fusion splicer. Moreover, based on these results can be determined that the cavity length of the air microcavity is not changed in a sensitive way if 100 < *L* < 350 μm and *ND* = 7. However as was stated before, for *L* values out of this range the air microcavity could not be formed for the way as the HCPCF collapses.

Furthermore it is possible to expect that another FPI reflection spectrum can be present due to multiple reflections occurring within the silica wall. This FPI pattern should have Δ*λ* ≈ 10.82 nm, this considering that the silica wall has a refractive index *n*_1_ ≈ 1.4682 and thickness *l*_2_ = 70 μm. This can be appreciated clearly in Figure 5(a,d), where less ‘intense’ peaks appear over the well-defined FPI fringes. This superposed FPI spectrum can be explained by the effect of multiple reflections occurring in the FPI mirrors [20–22], in this case within the silica wall. Furthermore, in order to these multiple reflections occurs within the silica wall it sides must be quasi parallel [20,23] at its central region.

### 4.3. Characterization of the IFPI Spectral Response Considering Different Number of Arc Discharges

**Figure 6.**
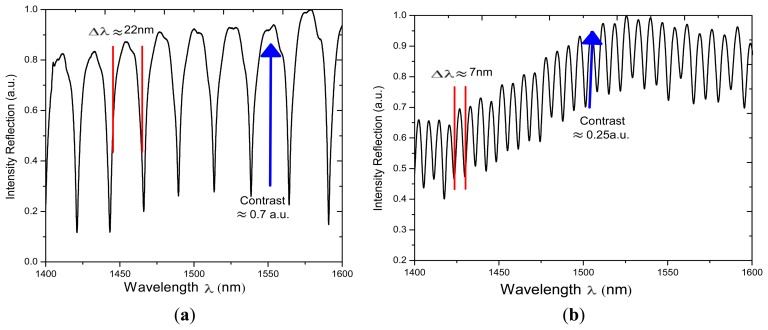
Measured spectrum reflection of two IFPI fabricated using *L*= 200 μm and (**a**) *ND* = 8; (**b**) *ND* = 12.

In order to characterize the effects of applying different number of arc discharges (ND) on the spectral response other two IFPI were fabricated. For this two *L* was fixed to 200 μm while for one we IFPI a *ND* = 8 was used and for the other one *ND* = 12 was used. The measured spectra of these two IFPIs are shown in Figure 6. Here can be appreciated that the free spectral range for the first IFPI is Δ*λ* ≈ 22 nm while for the second one is Δ*λ* ≈ 7 nm. This means that the length of the microcavity can be varied by applying different number of arc discharges (using the splicer program 2) to the HCPCF during the fabrication process. Moreover the fringe contrast is reduced when the optical distance is increased, which can be explained by the fact that the beam exiting from the SMF is diverging and this will strongly affect the contrast and the shape of the FPI fringes [20,24–26].

## 5. Potential Applications

**Figure 7.**
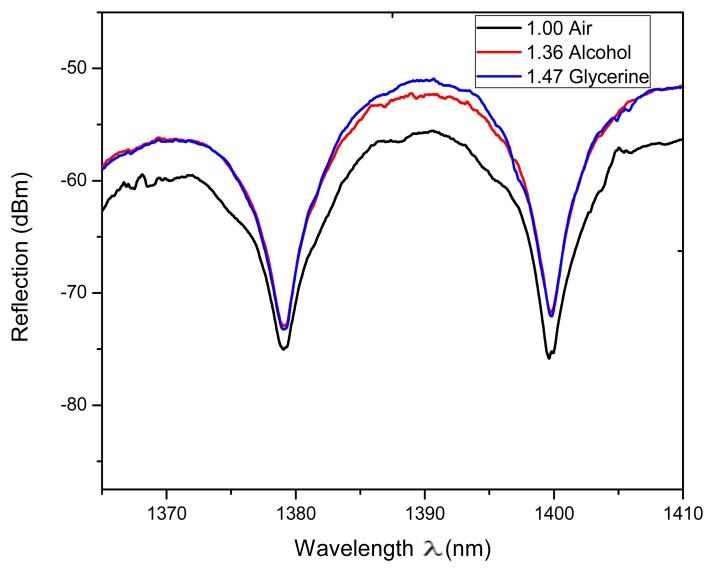
Measured reflection spectra of the IFPI immersed in different materials.

**Figure 8.**
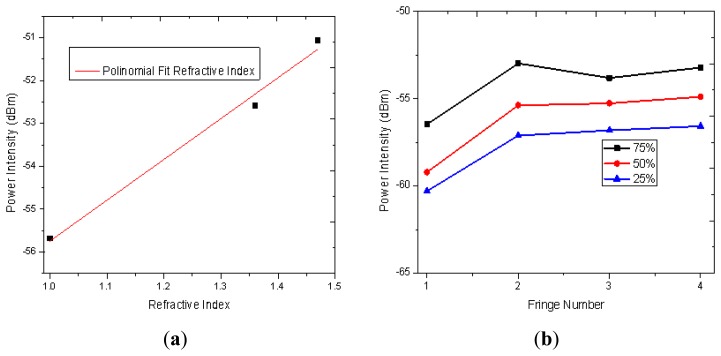
Refractive Index and Fringe contrast response: (**a**) Refractive index contrast intensity variations; (**b**) Fringe contrast variations from alcohol and water concentrations.

Some setups based on the FPI have been proposed to measure RI [27–30] which are useful in several areas such as in the wine industry and in biochemical applications. Hence to study the viability of our IFPI to implement a RI sensor the refractive index of the medium around it was varied (*n_3_*) and its effect on the spectral response characterized. For instance a detail of the measured reflection fringe pattern, from 1,380 to 1,400 nm, is shown in Figure 7. Here can be seen that the fringes were not shifted as *n_3_* was varied however the fringe contrast is affected. In our case the measured fringe contrast variation was in the order of 4.7 dBm when the RI was changed within a range from 1 to 1.473 (see Figure 8(a)).

In order to characterize the response of the IFPI to different concentrations of the same material some mixtures were prepared. For instance an IFPI was immersed in a mixture of 25%, 50% and 75% ethyl alcohol (*n* = 1.37) balanced with distillated water (*n* = 1.33) and the respective measured spectra from 1,360 to 1,460 was analysed (see Figure 8(b)). In this range it can be observed how the fringe peaks are ‘deeper’ as the concentration of the ethyl alcohol is increased. Based on these results it is possible to implement a RI based on the IFPI.

## 6. Conclusions

In this work a method to fabricate and IFPI based on an air microcavity was presented. It was shown that the fabrication method proposed provides flexibility to change the length of the air microcavity as well as it is low cost since requires the use of standard equipment. Moreover the spectral response of the IFPI and the air microcavity were characterized. Moreover it was shown that the spectral response of the fabricated IFPI is affected by changes of the medium around it. This is very important since it can be used to implement a simple and low cost refractive index sensor.

## Figures and Tables

**Table1.** Set of Parameters for Splicer Programs 1 and 2.

**Splicer Parameters**	**Program 1**	**Program 2**
Arc Power	2	254
Pre-fusion Time (ms)	50	110
Arc Duration (ms g)	50	750
Cleaning Time (ms)	200	200
Z-Push Distance (μm)	11	11
